# Supplementary figures and images for: The minus-end depolymerase KIF2A drives flux-like treadmilling of γTuRC-uncapped microtubules
Source: J Cell Biol. 2023 Aug 24;222(10):e202304020. doi: 10.1083/jcb.202304020 (PMC10450741; doi:10.1083/jcb.202304020)

**A, C** mScarlet-KIF2A / mCherry-MCAK

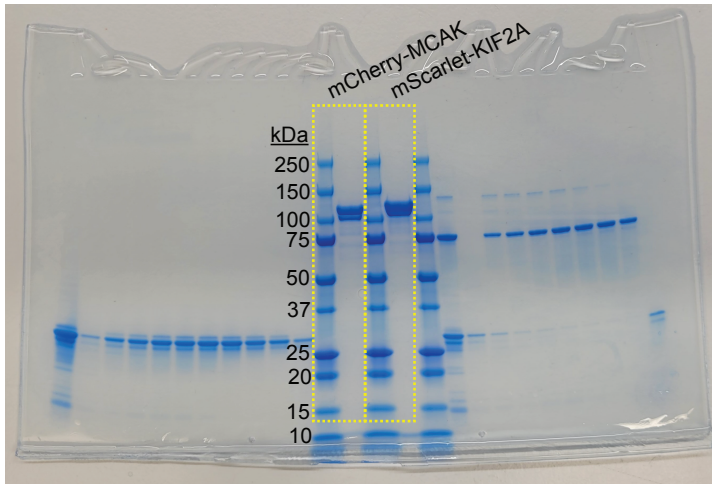

**F** spastin

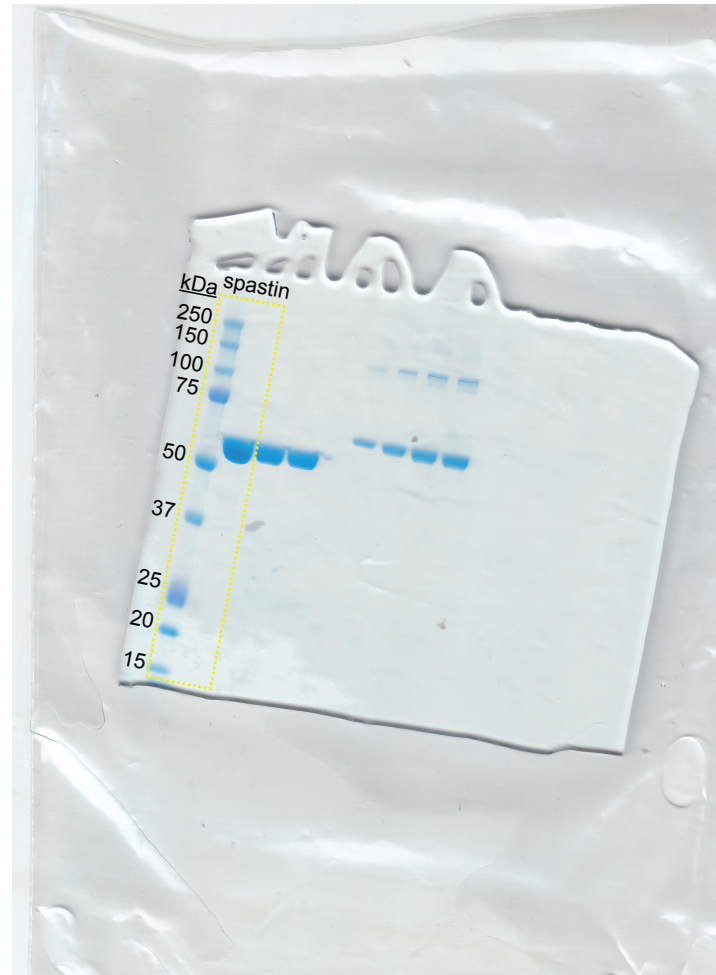

**B** untagged KIF2A

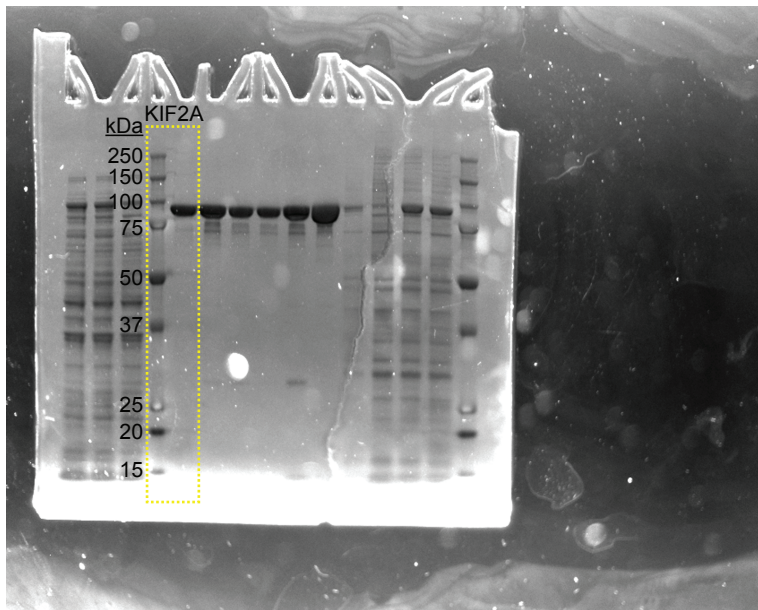

**D, G** iE5 / mGFP-spastin

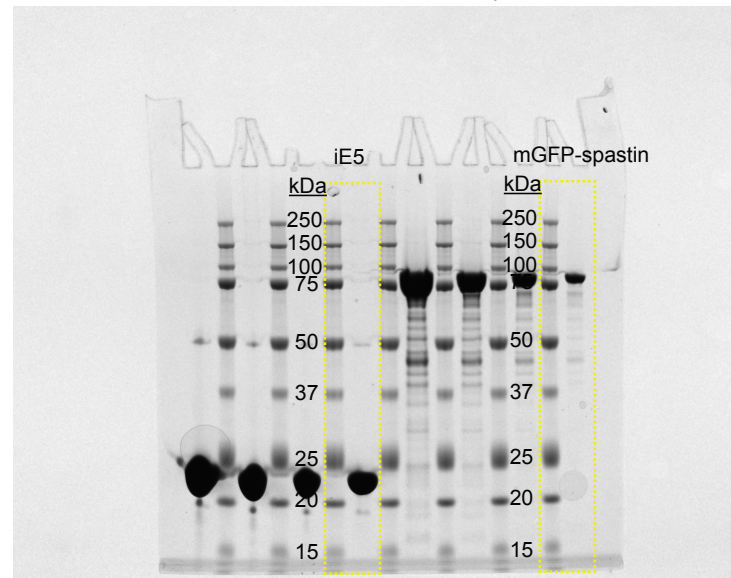

**E** DARPin (D1)<sub>2</sub>

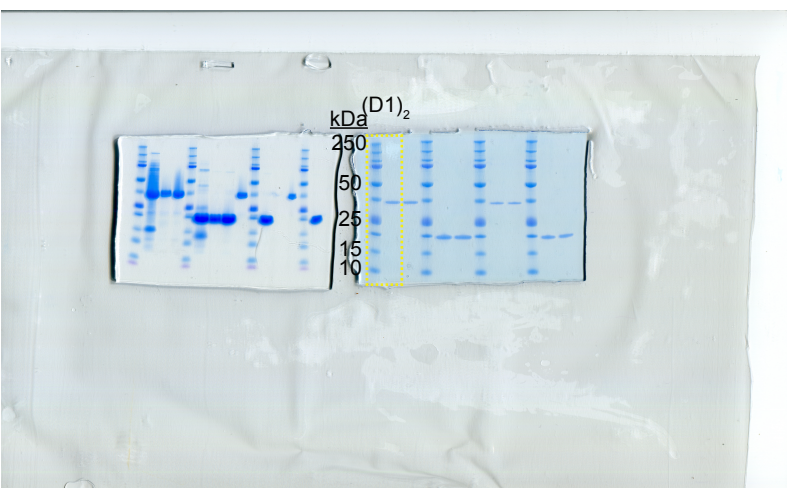

Supplement: SourceData FS1 — is the source file for Fig. S1. [file JCB_202304020_SourceDataFS1.pdf]
